# Total flavonoids of *Chrysanthemum indicum L* inhibit acute pancreatitis through suppressing apoptosis and inflammation

**DOI:** 10.1186/s12906-023-03851-x

**Published:** 2023-01-28

**Authors:** Xiaojuan Yang, Yun Liu, Chao Zhong, Jia Hu, Song Xu, Ping Zhang, Ling He

**Affiliations:** 1 Department of digestive system, Affiliated Hospital of Jiangxi University of Traditional Chinese Medicine, No. 445, Bayi Avenue, Nanchang, 330000 Jiangxi Province China; 2 Center of digestive endoscopy, Affiliated Hospital of Jiangxi University of Traditional Chinese Medicine, No. 445, Bayi Avenue, Nanchang, 330000 Jiangxi Province China

**Keywords:** NF-κB, MPO, TFC, Cerulein, Pancreatitis

## Abstract

Acute pancreatitis (AP) is one of the most common acute abdomen. Inflammation and apoptosis are closely linked with AP development. Total flavonoids of *Chrysanthemum indicum L* (TFC) has been proved to inhibit inflammation and apoptosis. If TFC could suppress AP remains unclear. AP animal and cell models were established with Cerulein. The pancreatic tissue injury was measured with HE staining. Inflammatory factors were detected with ELISA method. The protein expression was evaluated with Western blotting. Inhibition of AP in vivo was achieved by TFC by inhibiting serum amylase, myeloperoxidase (MPO), and water content of pancreatic tissue. The increased inflammatory response and activation of NF-κB signaling pathway in AP rats were inhibited after TFC treatment. The activation of NF-κB signaling pathway, increase of cell apoptosis and inflammatory factors in AR42J cells were suppressed by TFC. We demonstrated that TFC could significantly inhibit AP through restraining serum amylase, MPO, water content of pancreatic tissue, inflammation levels, apoptosis, and NF-κB signaling pathway activation. This study might clarify the potential inhibition mechanism of TFC in AP development.

**Supplementary Information:**

The online version contains supplementary material available at 10.1186/s12906-023-03851-x.

## Introduction

The *Chrysanthemum indicum L* has a wide range of pharmacological effects, including broad-spectrum antibacterial, anti-virus, anti-inflammatory, heart protection, and scavenging oxygen free radical effects [1, 2]. Total flavonoids of *Chrysanthemum indicum L* (TFC) are the main active components in *Chrysanthemum indicum* L. Some studies have shown that the TFC has a certain inhibitory effect on *Staphylococcus aureus*, *Escherichia coli, diphtheria*, etc. in vitro. In addition, TFC shows certain anti-inflammatory effects [3, 4].

Acute pancreatitis (AP) is a common acute abdomen with rapid onset and dangerous condition [5]. In addition to local pathological damage, it is often accompanied by strong systemic inflammatory cascade reaction, which can even lead to multiple organ failure and even death [6]. Clinical studies show that the mortality rate of AP is as high as 20%, so it has important clinical value to control or reduce severe acute pancreatitis [7]. When AP causes damage to the intestinal barrier function, bacteria or endotoxins can shift through multiple ways, causing intestinal flora shift and enterogenous endotoxemia [8]. Secondary infection of pancreatic tissue could start systemic inflammatory response syndrome (SIRS), and further cause multiple organ dysfunction syndrome (MODS) [9]. It plays an important role in protecting the stability of the internal environment of the organism by reducing inflammatory reaction, preventing pathogenic bacteria and toxins in the intestine from passing through the intestinal wall to the outside of the intestine [10].

Studies have shown that the TFC can improve the immune function of immunosuppressed mice and inhibit bacteria [11, 12]. In addition, TFC can inhibit the production of prostaglandin E2 and leukotriene B4 in the peritoneal macrophages of rats, and significantly reduced tumor cell necrosis factor-α level and neutrophil phagocytosis [13, 14]. Therefore, TFC could effectively inhibit inflammation. TFC is expected to become a new means of treating AP through suppressing inflammation, and the potential mechanism needs to be explored.

During the occurrence and development of AP, nuclear factor kappa B (NF-κB) plays an important role [15]. NF-κB signaling pathway directly participates in the regulation of inflammatory mediators such as TNF- α and IL-1β [16]. In addition, NF-κB could promote the infiltration of neutrophils and other inflammatory cells, leading to inflammatory reaction. Therefore, inhibition of NF-κB might alleviate the condition of AP [17]. However, if TFC could regulate AP through targeting NF-κB has not been reported.

In this study, Cerulein was used to establish AP models both in vivo *and* in vitro. The influence of TFC on the improvement of AP was investigated. The inhibition of inflammation response and apoptosis was observed after TFC treatment in the AP model. This research unfolds the potential mechanism of TFC in inhibiting AP.

## Methods

### Establishment of acute pancreatitis animal model

The intraperitoneal injection of Cerulein was used to induce acute pancreatitis as described previously [18, 19]. Forty-five SD rats were randomly divided into three groups, sham group, AP group and AP + TFC group. All rats were fasted 12 hours before induction and continued to fasted 6 hours after operation. After laparotomy, the intestinal and biliary pancreatic ducts were turned over in rats in the group sham without any other treatments. In the group AP and AP + TFC, rats were intraperitoneally injected with Cerulein (50 μg/kg) for 7 consecutive times at an interval of 1 hour. After Cerulein induction, the rats in the group AP + TFC were treated with TFC (300 mg/kg, once every 12 h, for 3 consecutive days) by gavage. The rats in group sham and AP were treated with same amount of normal saline. The rats in the group AP and AP + TFC also received laparotomy 2 h after final Cerulein induction. The rats in each group were sacrificed to collect tissue and blood samples. The peripheral blood samples of rats were obtained by removing their eyeballs. TFC was purchased from Xi’an Ruihe Bioengineering Technology Co., Ltd. (#RH11042685, Xi’an, China).

### Cell culture and AP cell model establishment

Rat pancreatic acinar cell, AR42J (#CRL-1492, ATCC), was used in this study. Cell adherence culture at 5 cm × 5 cm cell culture bottle. RPMI 1640 medium was used for cell culture, in which 10% FBS and Antibic Antimycotic double antibody were added. The culture condition was 37 °C and the CO_2_ concentration was 5%.

1 × 10^6^ cells were cultured in a 6-well plate for 24 h. Cerulein was added to medium with the final concentration of 10 nmol/L. After 4 h, TFC (50 mg/L) was added to culture cells for 24 h. Then, the supernatant of cell culture and AR42J cells were collected for subsequent experiments.

### Flow cytometry

Cells (1 × 10^5^ cells) were collected and centrifuged at 800 rpm at room temperature for 5 min, and supernatant was discarded. The cells were washed twice with PBS, and 500 μ L binding buffer (10 mM HEPES, pH 7.4, 140 mM NaOH. 2.5 mM CaCl_2_) were added to cells. The suspended cells were added 5 μL Annexin V–FITC, and kept away from light at room temperature for 15 min. Then, 10 μL PI were added. Finally, flow cytometry was performed to assess cell apoptosis.

### Hematoxylin and eosin (HE) staining

The freshly obtained mouse pancreatic tissue was fixed in formalin solution for 24 h. The pathological sections were dried overnight at 60 °C. The slices were soaked in xylene for dewaxing. The slides were washed with 100, 95, 75 and 50% ethanol for hydration in turn. Then, the slices were immersed in PBS for 3 min. Cell nucleus was stained with hematoxylin for 3 min, and tap water was used to rinse slides. The slices were put into 75% hydrochloric acid ethanol differentiation solution for 10 s, and washed with tap water for 3 minutes. The slices were soaked in 95% ethanol for 2 min, and stained with eosin for staining for 30 s. 75, 95 and 100% ethanol were used to dehydrate slides. Soaking in xylene for 5 min was performed to make slices transparent. After sealing with neutral gum, dry at 60 °C overnight. The sections were observed under the microscope.

### Immunohistochemistry (IHC) staining

The slides were prepared as described in the part 2.4. The sections were treated with antigen repair by putting into a microwave oven for 3 min. 5% hydrogen peroxide solution was used to treat sections for blocking endogenous peroxidase. After washing with PBS, the slides were treated with 5% BSA for 20 min. Then, sections were incubated with primary antibody overnight at 4 °C. PBS was used to wash slides, and sections were incubated secondary antibody for 3 h. DAB regents were used to incubate slides. Sections were observed under an inverted light microscope and images were obtained.

### Detection of TNF-𝑎, IL-6, IL-1β, IL-10, and amylase

Detection of amylase, TNF-𝑎, IL-6, IL-1β, IL-10 in supernatant of AR42J cell culture and serum of rats was performed with ELISA methods according to the instructions of the kits. These kits were purchased from Beyotime (Shanghai, China).

### Western blotting

The proteins in pancreatic tissues and cells were extracted and then quantified by BCA method. SDS-PAGE gel electrophoresis was performed. The protein was transferred to the membrane. 5% skimmed milk was used for blocking at room temperature for 1 hour. Primary antibody (1:1000) was added to incubate membrane overnight at 4 °C. TBST was used for washing 3 times. HRP labeled secondary antibody (1:2000) was added to incubate protein at 37 °C for 2 hours. TBST was used for washing 3 times. Image J software was used to analyze protein bands. All antibodies were purchased form Abacm. The blots were cut prior to hybridisation with antibodies to save antibodies. Therefore, full-length blots are not provided in the supplementary file.

### RT-PCR

The pancreatic tissues were lysed, and RNA was extracted with Trizol regent (TaKaRa). RNA concentration was measured. Reverse transcription was performed to generate cDNA. The reaction condition was listed below: 95 °C (1 min), and 40 cycles of 95 °C (20 s), 56 °C (20 s), and 72 °C (38 s). 2^-△△Ct^ method was applied for gene expression analysis. The premiers were listed as follows: COX2: F: 5′- ACAAGCAGTGGCAAAGC -3′, R: 5′- AAAGAGGCGAAGGGACA − 3′; p65: F: 5′- CTGCAGTTTGATGATGAAG-3′, R: 5′- TAGGCGAGTTATAGCCTCAG-3′; GAPDH: F: 5′- GACTCCACTCACGGCAAAT -3′, R: 5′- GACTCCACGACATACTCAGCA -3′.

### Measurement of trypsin activity in pancreatic acinar cell

The cell culture and treatment with Cerulein and TFC were described in the part 2.2. Trypsin activity in the supernatant after cell homogenization was detected in strict accordance with the instructions of the trypsin activity test kit (#A080–2-2, Nanjing Jiancheng Bioengineering Institute, Nanjing, China). The measuring tube and blank tube both contain trypsin substrate reaction solution (1.5 mL) preheated at 37 °C. Then, supernatant samples (50 μL) were added to related tubes. After incubation at 37 °C for 30 s and 20 min, the absorbance values at 253 nm were measured. The trypsin concentration can be calculated according to the formula.

### Statistical analysis

SPSS22.0 statistical software was used for data analysis. The data were expressed as mean ± standard deviation. ANOVA analysis was used for comparison among multiple groups, and independent sample t-test was used for comparison between two groups. *P* < 0.05 means statistically significant difference.

## Results

### Inhibition of AP in vivo was achieved by TFC

**Fig. 1:**
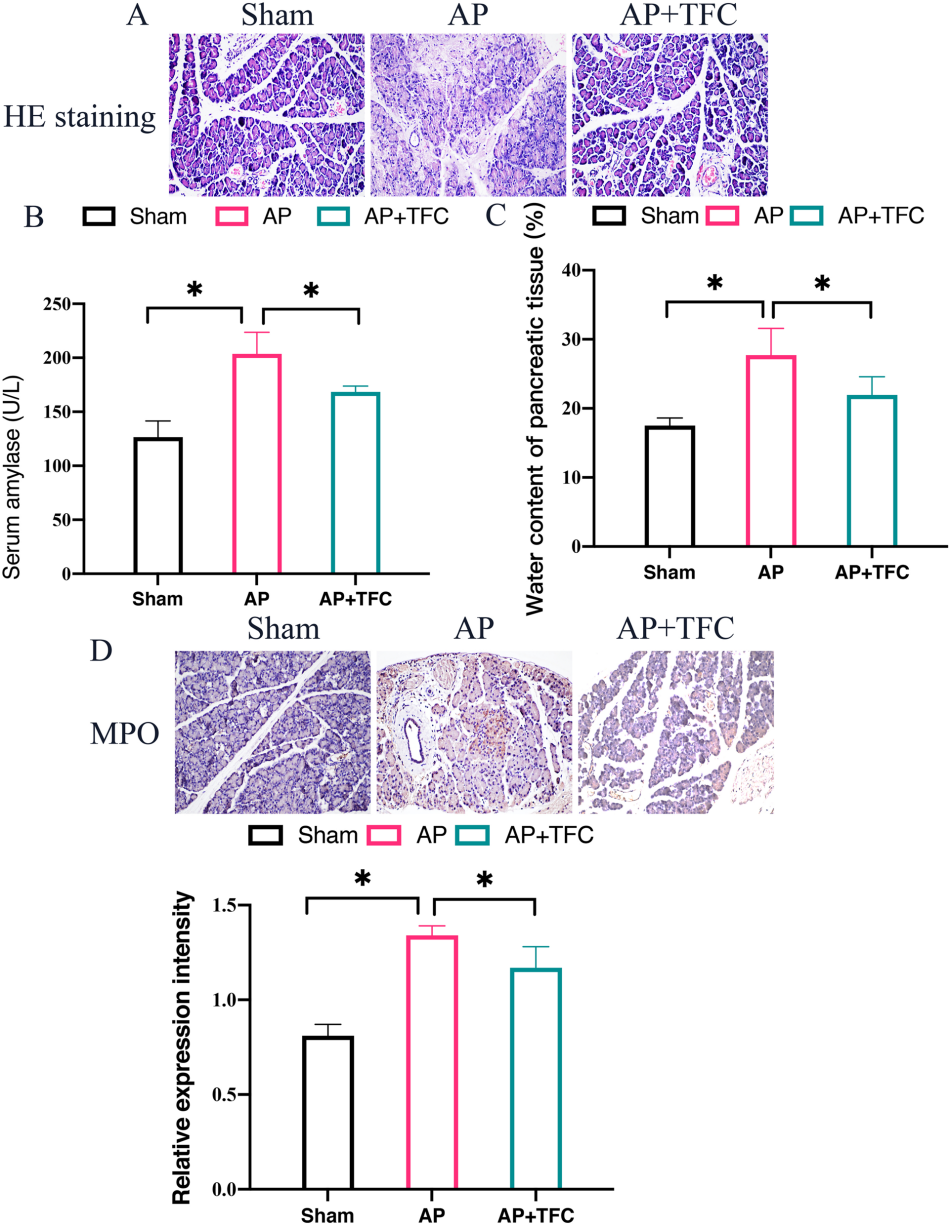
Inhibition of AP in vivo was achieved by TFC. **A** HE staining was performed to observe pancreatic tissue injury; **B** The level of serum amylase was measured with ELISA method; **C** The water content of pancreatic tissue was calculated; **D** The expression intensity of MPO in the pancreatic tissue was evaluated with IHC staining. * indicates *p* < 0.05

HE staining was performed to investigate the influence of TFC on pancreatic tissue injury. The pancreatic cells of rats in the sham operation group were structurally intact, no edema or inflammatory cell infiltration was found in the stroma. In AP group, a large number of pancreatic cells died, and a large number of inflammatory cells infiltrated into the stroma with edema. After TFC intervention, pancreatic cell death and inflammatory cell infiltration decreased significantly (Fig. 1 A). In addition, in the Cerulein treated rats, the level of serum amylase (Fig. 1 B), water content of pancreatic tissue (Fig. 1 C), and myeloperoxidase (MPO) expression intensity (Fig. 1 D) in the pancreatic tissue were greatly promoted. However, TFC treatment markedly inhibited these items suggesting that TFC might play a protective role against AP injury.

### The increased inflammatory response in AP were decreased after TFC treatment

**Fig. 2:**
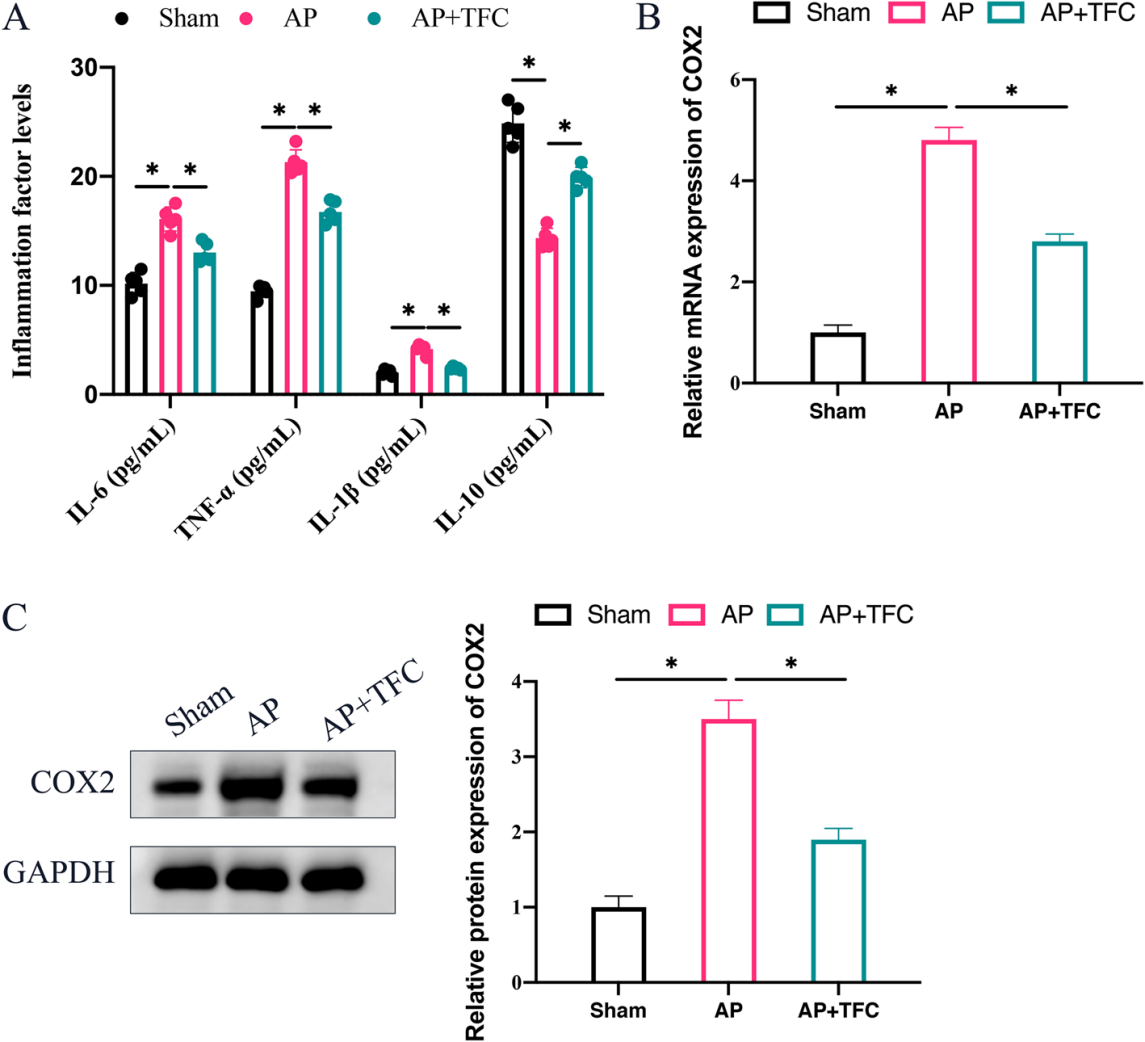
The increased inflammatory response in AP were decreased after TFC treatment. **A** The concentrations of IL-1β, TNF-𝑎, IL-10, and IL-6 in the serum were detected with ELISA method; **B** The mRNA expression level of COX2 was analyzed with RT-PCR; **C** The protein expression level of COX2 was detected with Western blotting. * indicates *p* < 0.05

In the group AP, the expression levels of pro-inflammatory factors including IL-1β, TNF-𝑎, and IL-6 were greatly promoted, but IL-10 was significantly decreased (Fig. 2 A). However, the changing trend of inflammatory factors in AP rats were reversed by TFC. Inflammatory damage can induce the production of COX2, which is the key link to trigger the subsequent inflammatory response. In the pancreatic tissues of AP rats, the mRNA and protein expression of COX2 were remarkably elevated (Fig. 2 B-C), but the level of COX2 were suppressed by TFC.

### The activation of NF-κB signaling pathway in AP rats was suppressed by TFC

**Fig. 3:**
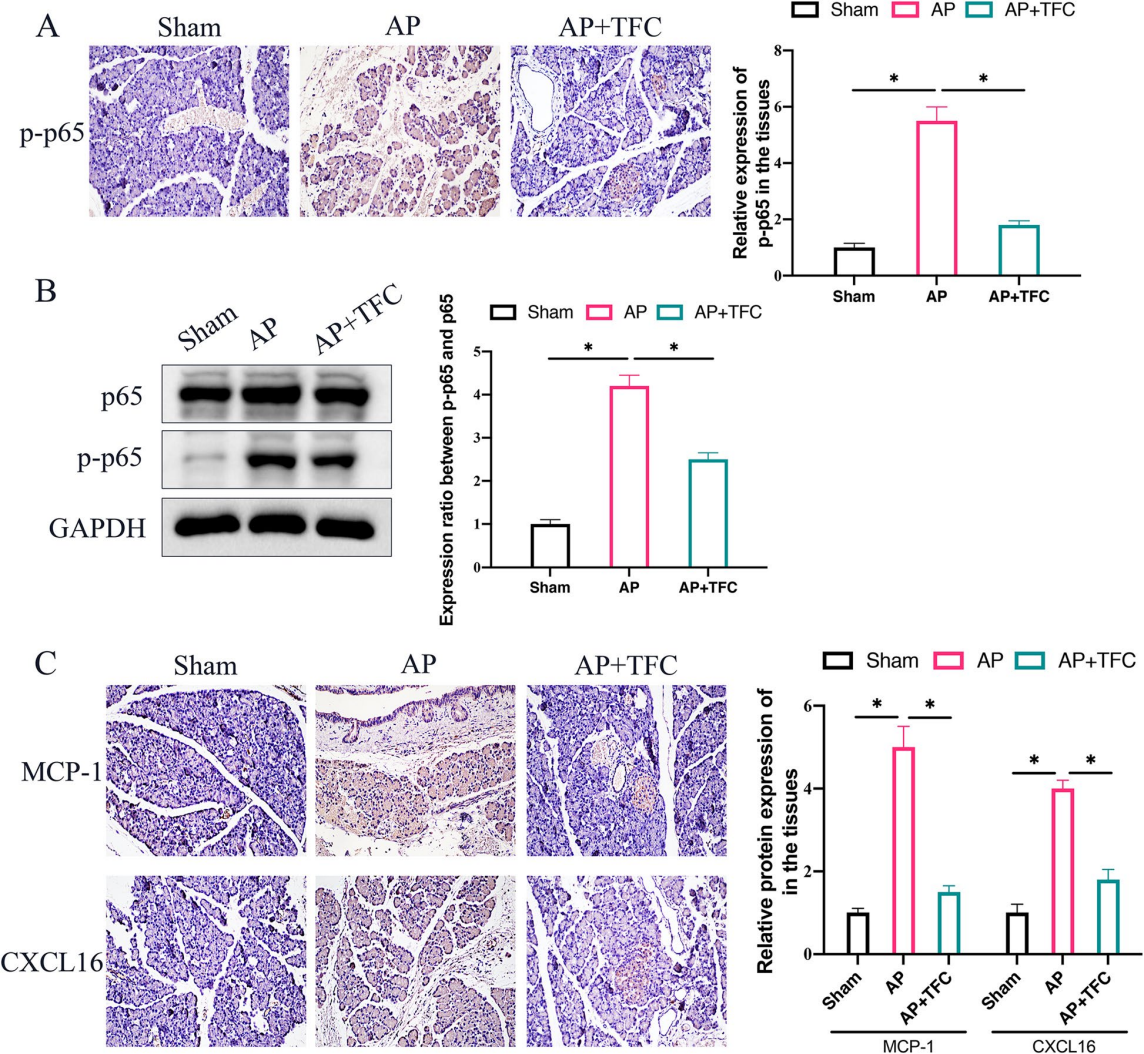
The activation of NF-κB signaling pathway in AP rats was suppressed by TFC. **A** The phosphorylation of p65 in the pancreatic tissues was evaluated with IHC staining; **B** The phosphorylation of p65 in the pancreatic tissues was measured with Western blotting method; **C** The protein levels of MCP-1 and CXCL16 in the pancreatic tissues was measured with IHC staining. * indicates *p* < 0.05

Phosphorylation of p65 (p-p65) was believed to be the marker of NF-κB signaling pathway activation. We found that the expression of p-p65 was remarkably promoted in the pancreatic tissues of AP rats (Fig. 3 A-B) measured by IHC staining and Western blotting. However, the level of p-p65 was greatly inhibited after TFC treatment, suggesting that TFC could restrain the activation of NF-κB signaling pathway. In addition, the regulatory role of chemokines including MCP-1 and CXCL16 in AP has been reported. We found that the levels of MCP-1 and CXCL16 in Cerulein-induced AP were greatly elevated (Fig. 3 C). However, TFC treatment remarkably suppressed the expression of MCP-1 and CXCL16.

### The increase of cell apoptosis and inflammatory factors in vitro were suppressed by TFC

**Fig. 4:**
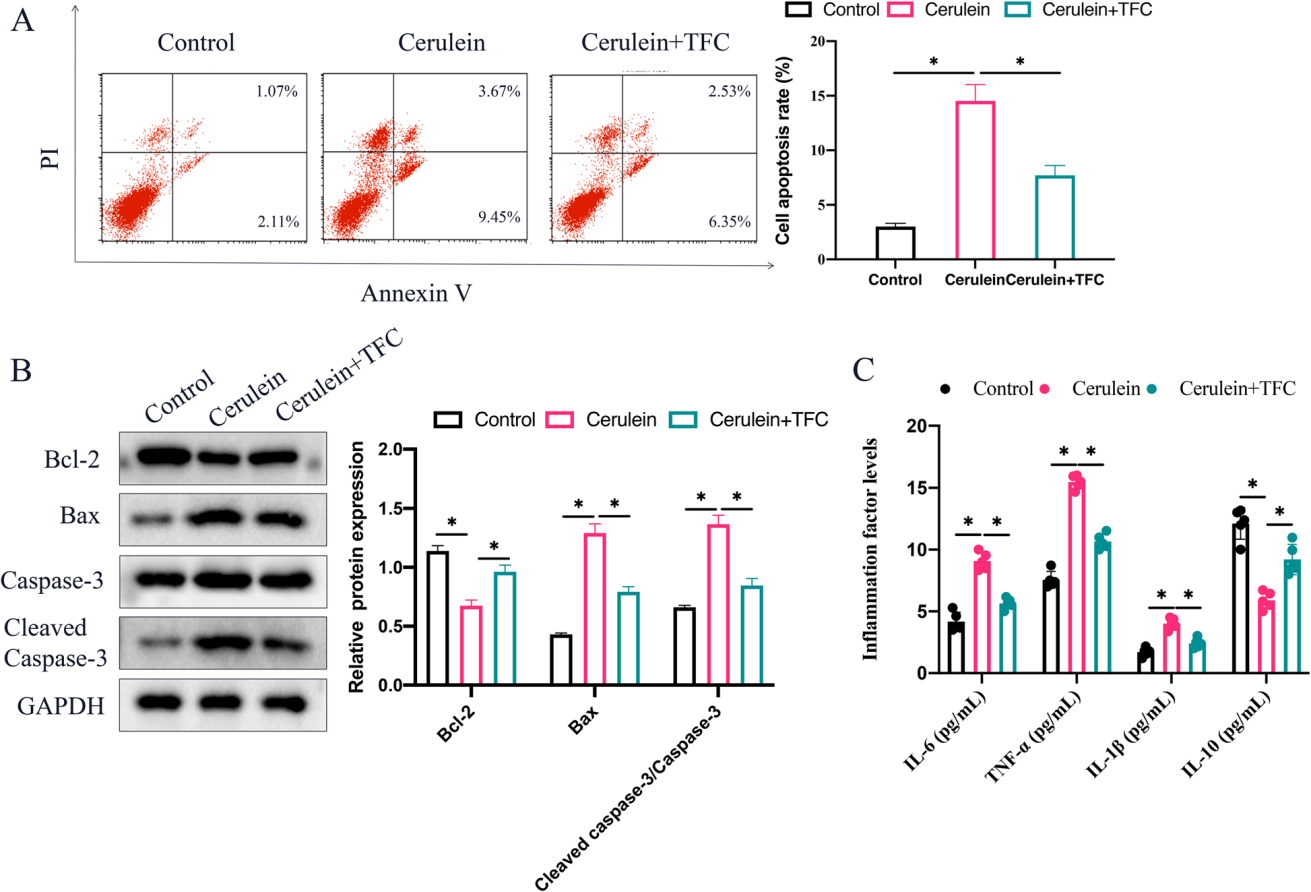
The increase of cell apoptosis and inflammatory factors in vitro were suppressed by TFC. **A** Cell apoptosis was measured with flow cytometry; **B** Apoptosis related proteins were measured with Western blotting; **C** The levels of inflammation related factors were detected with ELISA. * indicates *p* < 0.05

Cerulein was used to treat AR42J cells to simulate AP in vitro model. After cerulein, significant increase of cell apoptosis (Fig. 4 A) and pro-apoptotic genes (Bax and cleased caspase-3), but remarkable decrease of Bcl-2 (Fig. 4 B) were observed compared with group control. However, TFC treatment greatly reversed the effects of cerulein, and suppressed cell apoptosis rate (Fig. 4 A-B). Meanwhile, TFC also greatly restrained the increased levels of inflammation factors induced by cerulein (Fig. 4 C).

### The activation of NF-κB signaling pathway in cells was suppressed by TFC

**Fig. 5:**
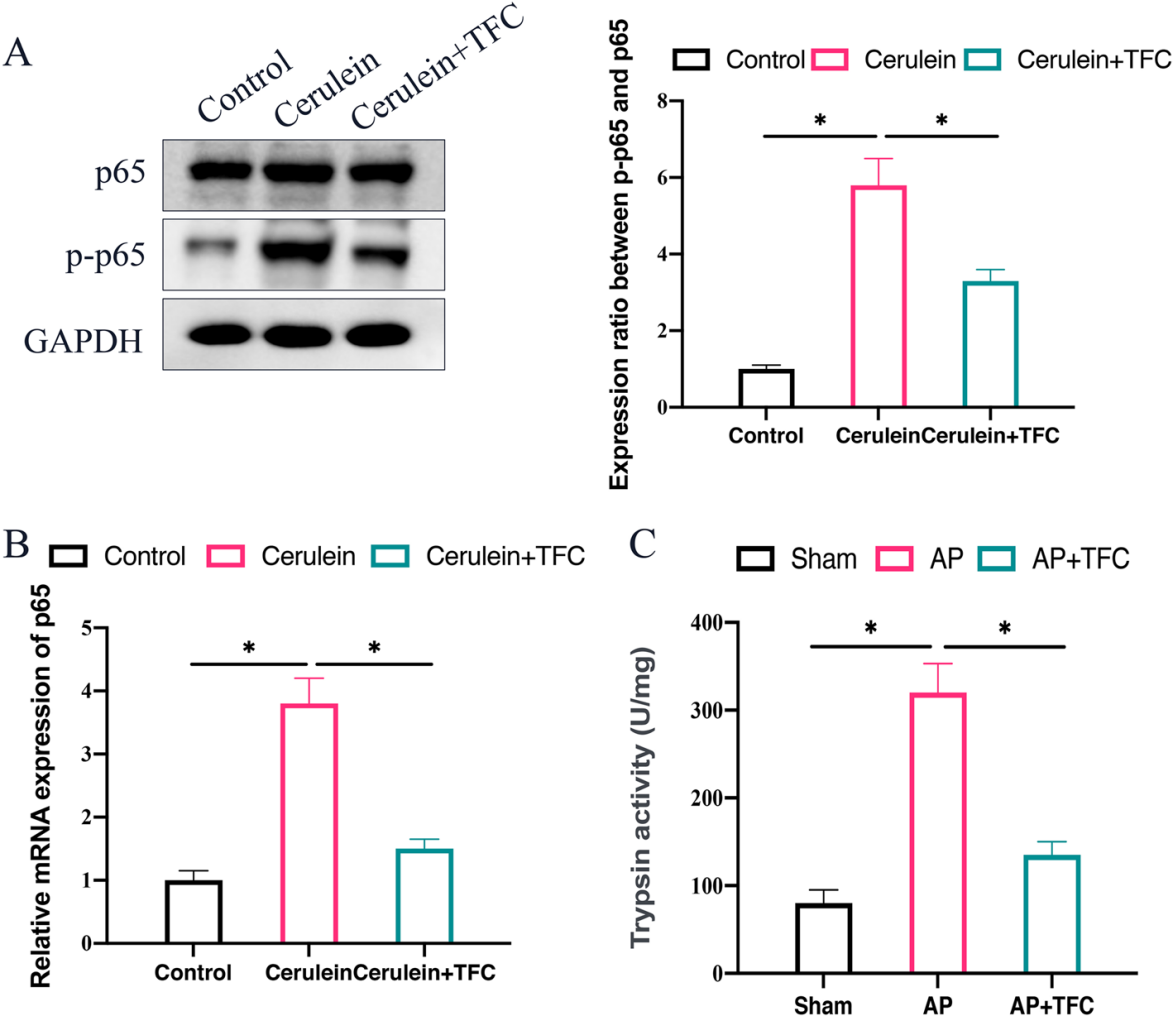
The activation of NF-κB signaling pathway in cells was suppressed by TFC. **A** The phosphorylation of p65 in AR42J cells was measured with Western blotting method; **B** The mRNA level of p65 in AR42J cells was measured with RT-PCR; **C** The trypsin activity in the pancreatic acinar cell was detected. * indicates *p* < 0.05

The influence of TFC on NF-κB signaling pathway activation was also validated in vitro. We found that cerulein could also increase the p-p65 expression in AR42J cells (Fig. 5A-B). However, the activation of NF-κB signaling pathway was suppressed by TFC through inhibiting p-p65. Meanwhile, the significant increase of trypsin activity in Cerulein-treated pancreatic acinar cell was greatly inhibited after TFC treatment (Fig. 5 C).

## Discussion

AP is a common clinical acute abdomen, which belongs to SIRS. AP is mostly related to bacterial translocation caused by the destruction of intestinal mucosal barrier. It has been found that the impairment of intestinal mucosal barrier function caused by microcirculation disorder, ischemia reperfusion injury, excessive release of inflammatory mediators and apoptosis can aggravate AP.

*Chrysanthemum indicum* is a kind of herbaceous plant, which is distributed in most areas of China and is very rich in resources [20]. Modern pharmacological studies show that *Chrysanthemum indicum* has broad-spectrum antibacterial activity [3]. It was reported that the TFC have significant anti-inflammatory and analgesic activities [3]. However, the regulation of TFC during the progression of AP has not been fully clarified. In this study, we firstly demonstrated that TFC inhibited AP through suppressing inflammation, apoptosis, and activation of NF-κB. AR42J cell line is derived from rat pancreatic acinar cell tumor. AR42J cells retain pancreatic exocrine characteristics and can secrete a variety of digestive enzymes, so they are widely used to build AP cell models [5]. In this research, the remarkable increase of apoptosis, inflammatory response, and NF-κB signaling pathway activation in AR42J cells was greatly restrained by TFC treatment.

In the development of AP, TNF-α plays an important role in promoting the production of other inflammatory factors [21]. TNF-α can also activate NF-κB, and further cause a magnified inflammatory reaction [22]. Therefore, regulation of TNF-α could not only alleviate the symptoms of AP, reduce the release of inflammatory mediators, but also inhibit NF-κB activation [23]. In this study, we found that TFC markedly inhibited the production of TNF-α in both in vivo (Fig. 2 A) and in vitro (Fig. 4 C) levels.

Apoptosis is believed to be closely linked with the progression of AP [24]. Bax is an important apoptosis promoting factor in mitochondrial pathway, which can induce apoptosis [25]. Bcl-2 is an anti-apoptotic protein, which can not only regulate apoptosis, but also regulate autophagy. In addition, Bcl-2 can also regulate oxidative stress induced autophagy and apoptosis [26]. Bcl-2 can competitively combine with Bax, neutralize Bax and reduce apoptosis [5]. We demonstrated that pro-apoptotic proteins were inhibited by TFC, and anti-apoptotic protein was promoted by TFC in pancreatic tissues and AR42J cells. Inhibition of apoptosis by TFC might be the potential regulatory mechanism in suppressing AP. However, the specific targeting signaling pathway of TFC improving AP and inactivating p-p65 needs to be further investigated, which is a limitation of this research.

## Conclusion

In vivo level, we found that TFC could significantly improve pancreatic tissue injury, inhibited serum amylase, MPO, water content of pancreatic tissue, inflammatory response, and NF-κB signaling pathway activation. In vitro level, inhibitions of cell apoptosis, inflammatory factors expression, and NF-κB signaling pathway activation were validated. This study unfolds the potential inhibition mechanism of TFC in AP development.

## Supplementary Information

**Additional file 1.**

## Data Availability

The datasets used in the current study are available from the corresponding author on reasonable request.

## Abbreviations

AP: Acute pancreatitis
TFC: Total flavonoids of *Chrysanthemum indicum L*
MPO: Myeloperoxidase
SIRS: Systemic inflammatory response syndrome
MODS: Multiple organ dysfunction syndrome
IHC: Immunohistochemistry
HE: Hematoxylin and eosin
NF-κB: Nuclear factor kappa B
