# Supplementary material for: Total flavonoids of Chrysanthemum indicum L inhibit acute pancreatitis through suppressing apoptosis and inflammation
Source: BMC Complement Med Ther. 2023 Jan 28;23:23. doi: 10.1186/s12906-023-03851-x (PMC9883918; doi:10.1186/s12906-023-03851-x)

Figure 2 C

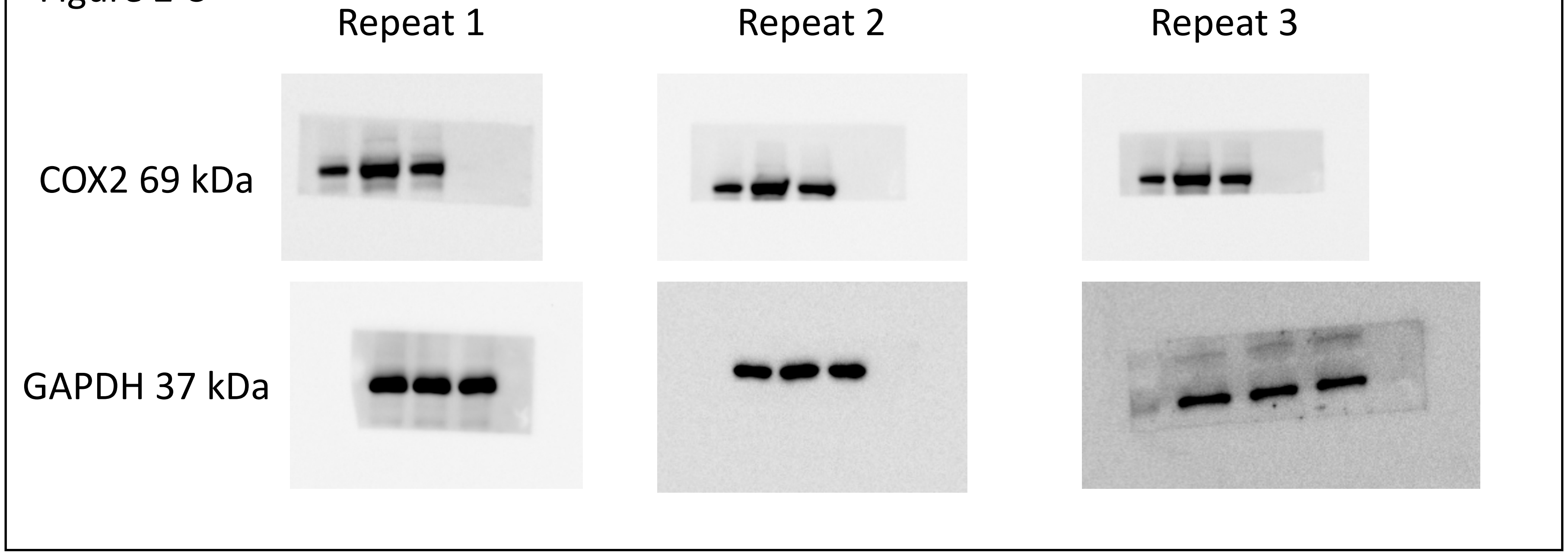

Figure 3 B

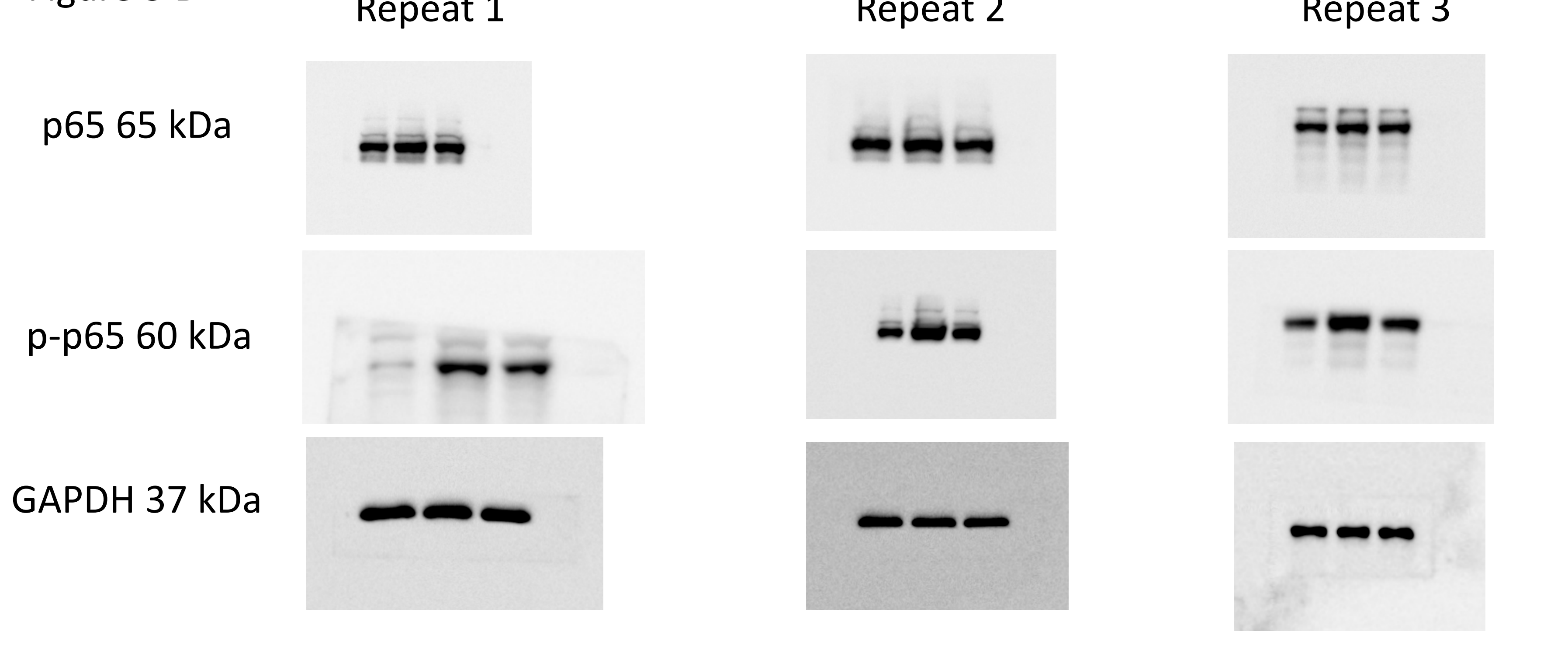

Figure 4 B

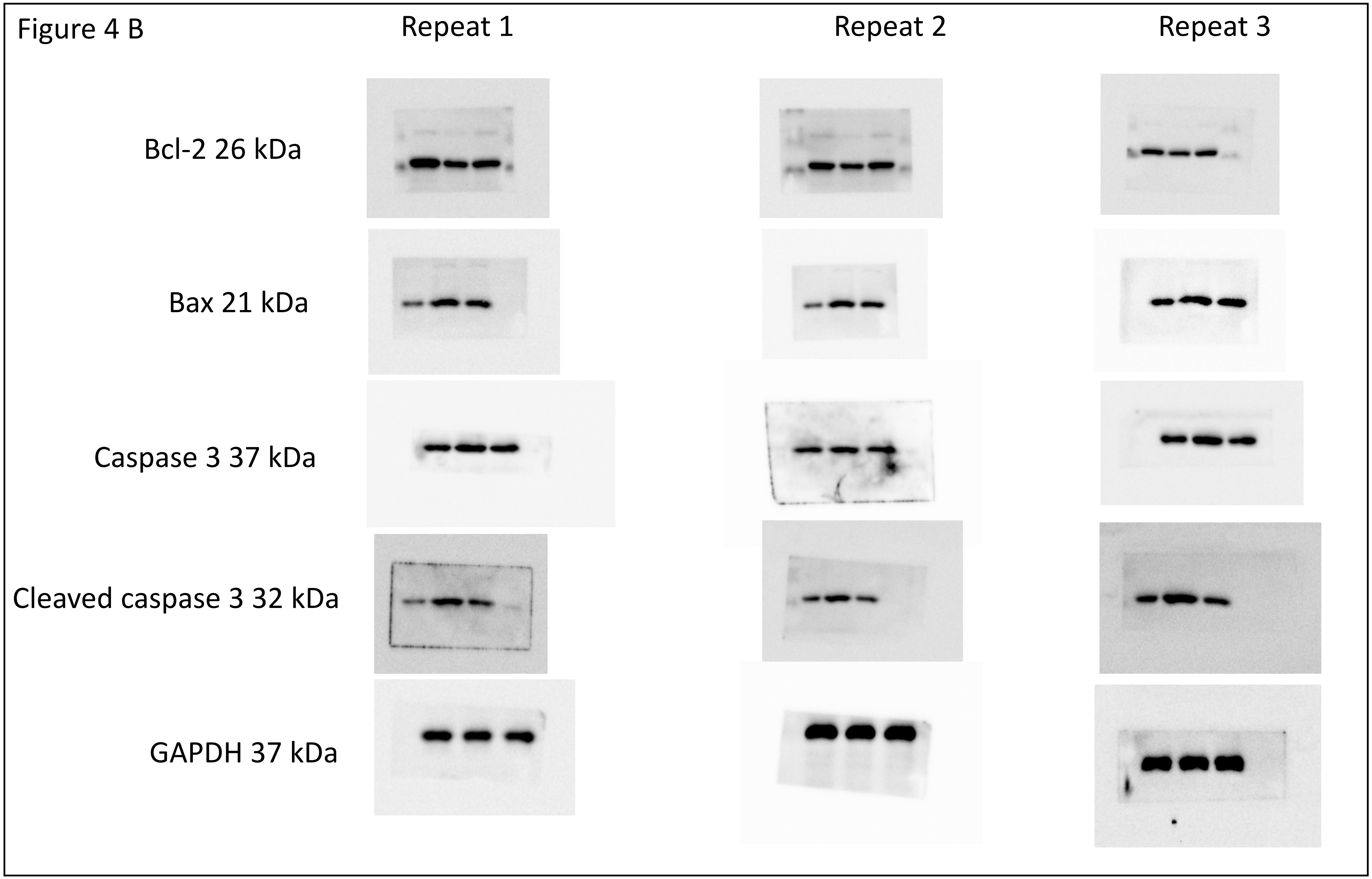

Repeat 1
Repeat 2
Repeat 3
Bcl-2 26 kDa
Bax 21 kDa
Caspase 3 37 kDa
Cleaved caspase 3 32 kDa
GAPDH 37 kDa

Figure 5 A

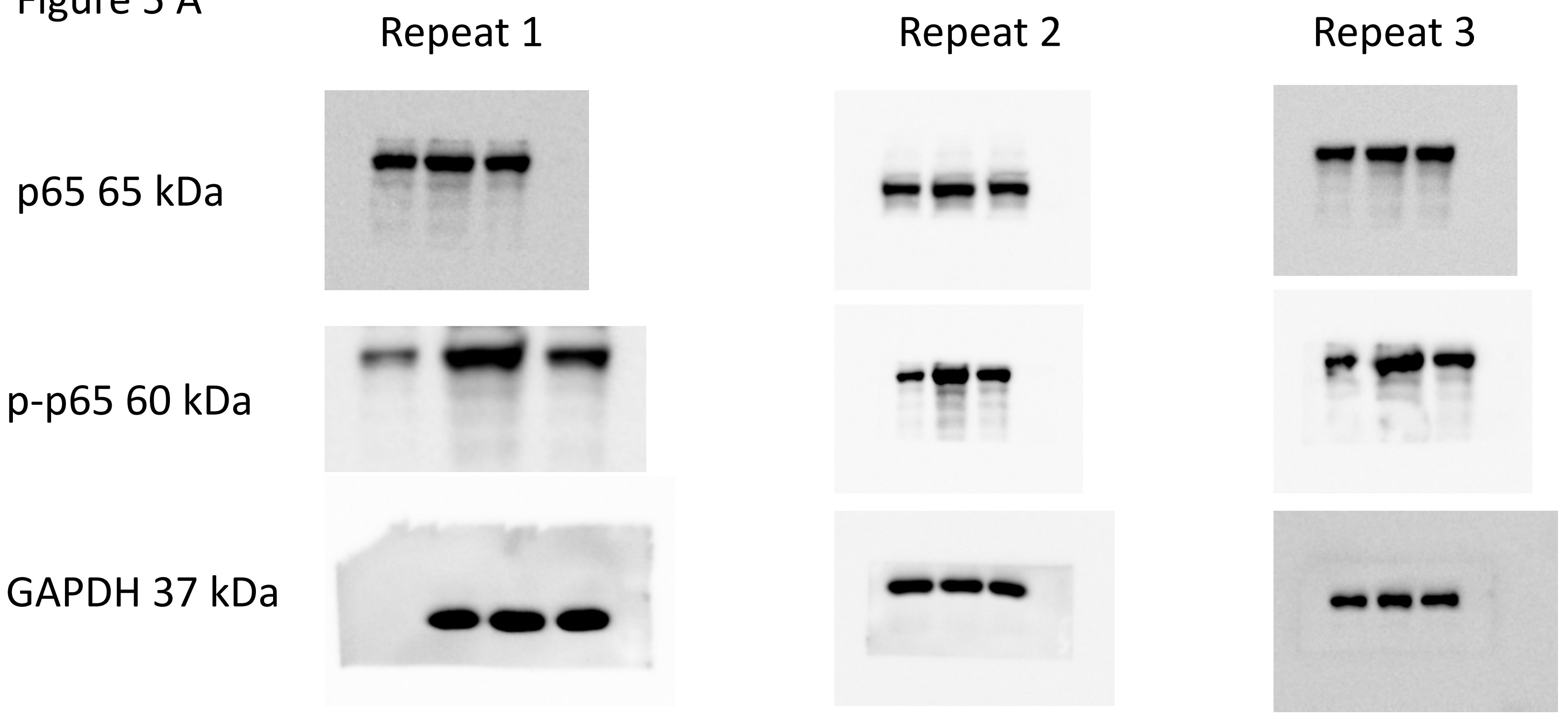

Supplement: Supplementary file 1 — Additional file 1. [file 12906_2023_3851_MOESM1_ESM.pdf]
